# Natural flavones from edible and medicinal plants exhibit enormous potential to treat ulcerative colitis

**DOI:** 10.3389/fphar.2023.1168990

**Published:** 2023-06-01

**Authors:** Qiang Lu, Yuhong Xie, Jingbin Luo, Qihai Gong, Cailan Li

**Affiliations:** ^1^ Department of Pharmaceutical Sciences, Zhuhai Campus, Zunyi Medical University, Zhuhai, China; ^2^ Department of Pharmacology, Zhuhai Campus, Zunyi Medical University, Zhuhai, China; ^3^ China Traditional Chinese Medicine Holdings Company Limited, Foshan, China; ^4^ Key Laboratory of Basic Pharmacology of Ministry of Education and Joint International Research Laboratory of Ethnomedicine of Ministry of Education, Zunyi Medical University, Zunyi, China; ^5^ Key Laboratory of Basic Pharmacology of Guizhou Province and School of Pharmacy, Zunyi Medical University, Zunyi, China

**Keywords:** ulcerative colitis, natural flavones, edible and medicinal plants, therapeutic effect, mechanism

## Abstract

Ulcerative colitis (UC) is a chronic aspecific gut inflammatory disorder that primarily involves the recta and colons. It mostly presents as a long course of repeated attacks. This disease, characterized by intermittent diarrhoea, fecal blood, stomachache, and tenesmus, severely decreases the living quality of sick persons. UC is difficult to heal, has a high recurrence rate, and is tightly related to the incidence of colon cancer. Although there are a number of drugs available for the suppression of colitis, the conventional therapy possesses certain limitations and severe adverse reactions. Thus, it is extremely required for safe and effective medicines for colitis, and naturally derived flavones exhibited huge prospects. This study focused on the advancement of naturally derived flavones from edible and pharmaceutical plants for treating colitis. The underlying mechanisms of natural-derived flavones in treating UC were closely linked to the regulation of enteric barrier function, immune-inflammatory responses, oxidative stress, gut microflora, and SCFAs production. The prominent effects and safety of natural-derived flavones make them promising candidate drugs for colitis treatment.

## Introduction

Ulcerative colitis (UC) is a major type of inflammatory bowel disease, which has been widely prevalent all over the world (Alsoud et al., 2021). Traditionarily, UC is considered to be a common Western disorder (Ge et al., 2021). It was announced that the yearly morbidity of colitis in European countries is as high as 0.0243%, and that in Northern America is up to 0.0192% (Du and Ha, 2020). By comparison, the annual morbidity of UC in Asian nationalities is fairly lower, at less than 0.01% (Wei et al., 2021). Whereas, in recent years, due to the changes in life and environment, the annual incidence rate of UC among Asian nationalities has been rising steadily over time (Hirten and Sands, 2021; Zeng et al., 2022). Consequently, this disease brought great pressure to the financial and medical services of all countries.

UC is a chronic nonspecific inflammatory disorder of the colon and rectum with uncertain aetiology (Sinopoulou et al., 2021). The lesion is confined to the mucosal and submucosal layers of intestinum crassum. Furthermore, the majority of lesions are found in the rectum and sigmoid colon, but they can also extend to the descending colon or the whole colon (Li et al., 2022). UC has a long course and often occurs repeatedly. Moreover, this disease occurs at any age, although it most frequently strikes people between the ages of 20 and 40 (Li C. L. et al., 2021). Although the aetiology of UC is uncertain, it is generally accepted that genes are an important cause of colitis. Psychological factors play an important role in the deterioration of UC. Following colectomy, the original morbid spirit, such as depression or social isolation, is obviously improved. In addition, it is also recognized that colitis is an autoimmune disease (Fiorino et al., 2021).

**FIGURE 1:**
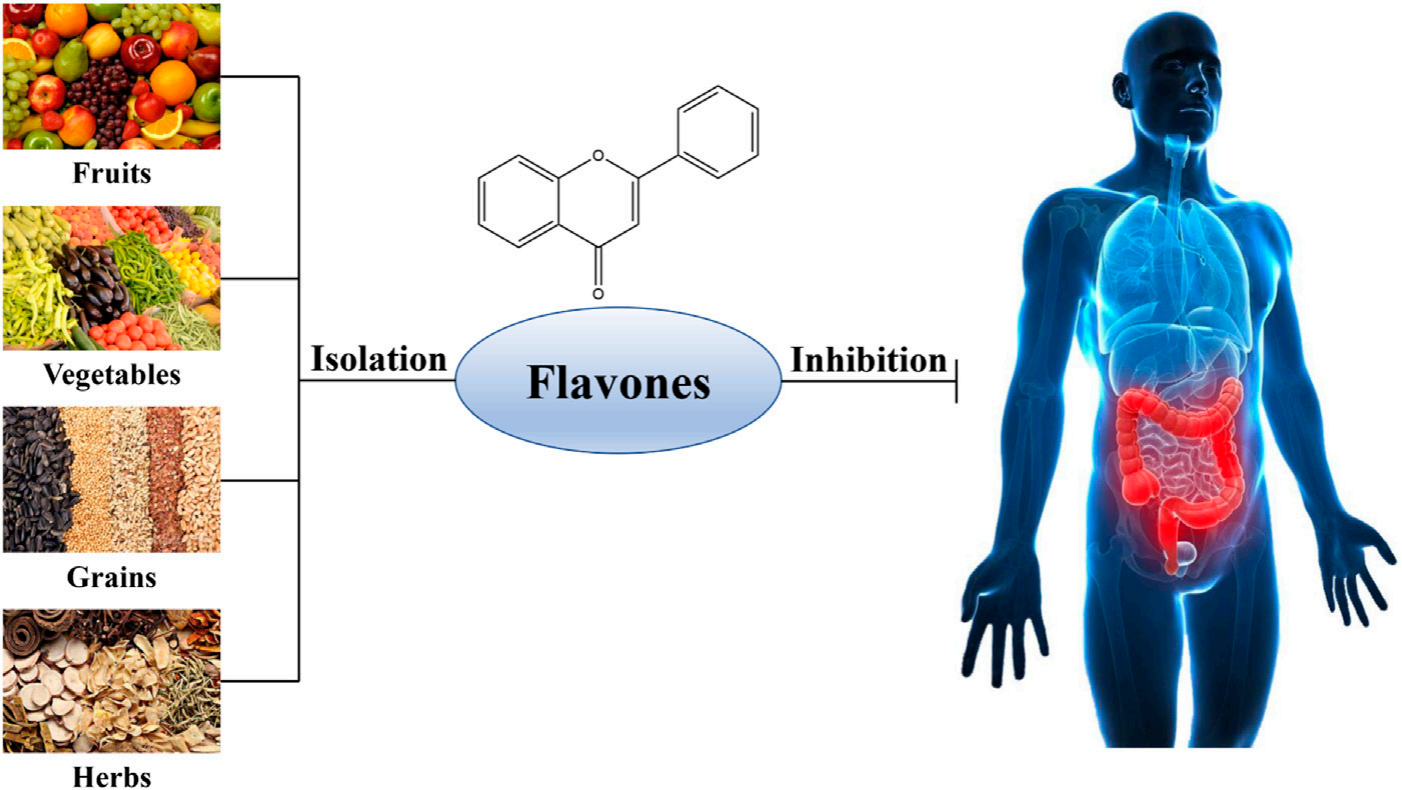
Natural sources of flavones against ulcerative colitis.

To date, there are still no satisfactory clinical drugs for colitis in terms of efficacy and safety. Nowadays, the drugs for colitis primarily consist of 5-amino salicylic acids, glucocorticoids and immunosuppressors, which lack specificity and are difficult to cure UC, accompanied by some adverse reactions, such as high recurrence, elevated resistance, etc. In this case, natural flavones raised extensive concerns as their no/low toxicity and excellent anti-inflammatory, antioxidative, antineoplastic, antibacterial, and immune-regulative activities in the prophylaxis and therapy of gastrointestinal diseases, tumors, and cardiovascular problems (Juca et al., 2020; Jeong et al., 2022). Numerous studies have testified that many kinds of flavones (Figure 1) from edible and medicinal plants possessed excellent therapeutic effects and safety in UC by multiple mechanisms involving ameliorating oxidative damage, reducing the inflammatory responses, preserving enteric barrier function, and adjusting gut flora structure, indicating that natural-derived flavones have the potentiality to suppress the progression of UC and put off the clinical course of this disease. Therefore, in this review, our team summarized the acting mechanisms of natural-derived flavones inhibiting colitis, such as apigenin, baicalein, diosmetin, sinensetin, wogonin, etc. These would provide new perspectives to develop effective medications for the management of colitis.

## Methods

For the sake of identifying the research connected with the therapeutic role and acting mechanism of natural flavones treating UC, our team conducted an extensive search of relevant articles in several databases, including Web of Science, PubMed, Elsevier, Google Scholar, and CNKI, covering the period from their inception until May 2023. We employed a combination of the following keywords during the literature retrieval: (“flavone” OR “flavonoid”) AND (“colitis” OR “colonitis” OR “UC”). All papers providing abstract will be considered.

After the search, the retrieved studies underwent a rigorous screened. Initially, studies on the therapeutic role and mechanisms of natural flavones treating UC were screened on the basis of titles and abstracts. For studies that were not able to be conclusively identified during the initial screening, their full-text versions were further evaluated. At last, all relevant studies including cell experiments, animal experiments and clinical trials were gathered and imported into EndNote software as support resources for the present review.

## Natural flavones against ulcerative colitis

**FIGURE 2:**
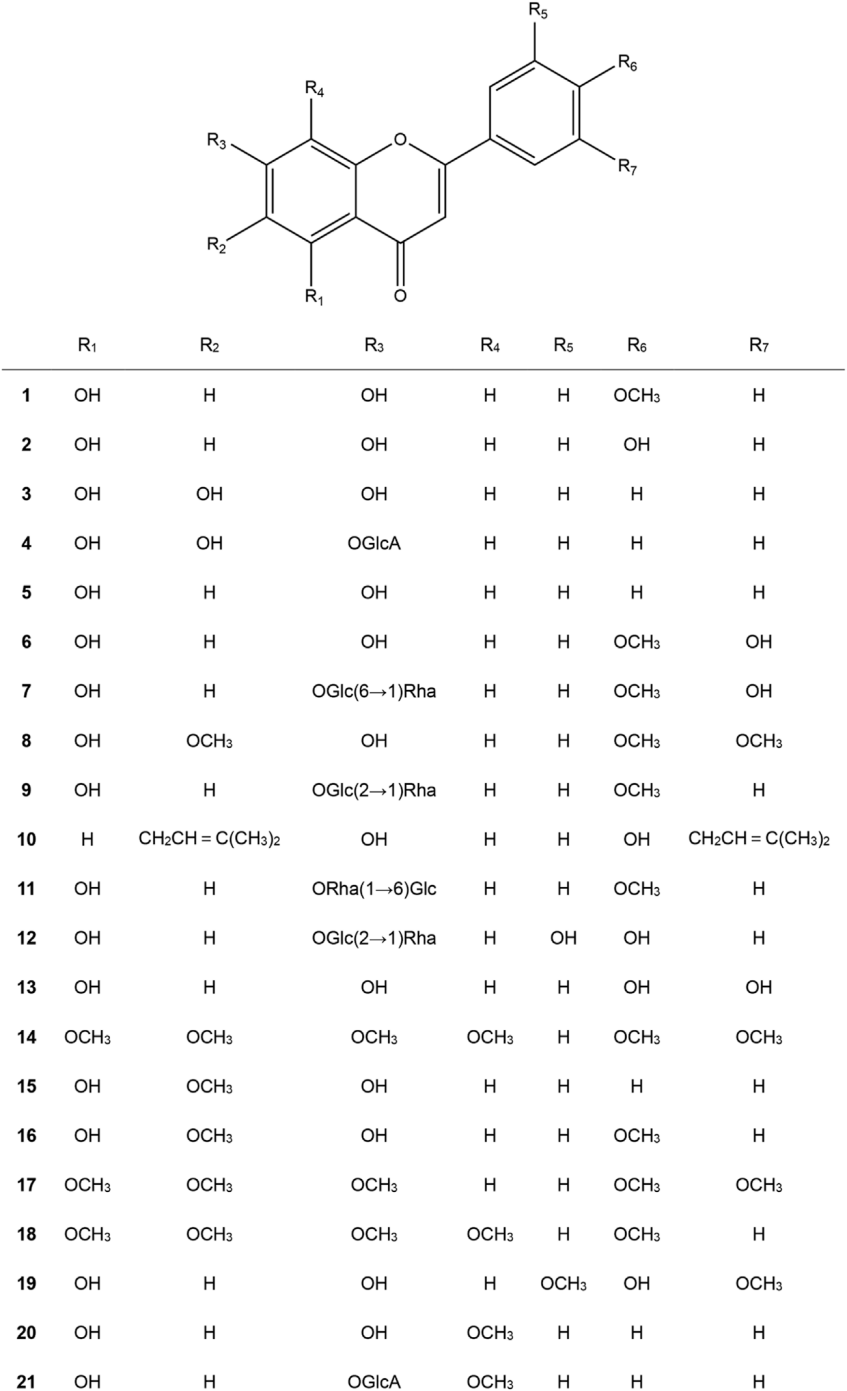
Chemical structures of naturally derived flavones for the treatment of ulcerative colitis.

The amount of identified flavonoids has risen to four thousand since the discovery of the first type of flavonoid vitamin P (namely, rutin), the amount of identified flavonoids has been up to four thousand. Of which, some possess powerful pharmacologic action and exhibit great potential in the exploitation of new medicines and their clinic practice. Based on the fundamental structure, currently known flavonoids are separated into seventeen classes, and flavones are the most important class and have received extensive attention. Therefore, the present review focused on natural-derived flavones for the treatment of UC. The general information of the included flavones is given in Table 1, while the chemical structures are presented in Figure 2. Additionally, Table 2 provides the pharmacological information of these flavones.

**TABLE 1:** Natural flavones separated from edible and medicinal plants.

No.	Flavones	Molecular formula	Molecular weight (g/mol)	Potential side effects	Main sources	References
1	Acacetin	C_16_H_12_O_5_	284.26	Cytochrome P450 inhibition	*Acacia farnesiana*; *Robinia pseudoacacia*; *Saussurea involucrata*; *Turnera diffusa*	Ren et al. (2021); Zhou et al. (2020)
2	Apigenin	C_15_H_10_O_5_	270.24	No	*Apium graveolens*; *Matiricaria recutita*; *Melissa axillaris*	Marquez-Flores et al. (2016)
3	Baicalein	C_15_H_10_O_5_	270.24	No	*Scutellaria baicalensis*; *Oroxylum indicum*; *Plantago major*	Li et al. (2021g); Liu et al. (2020a); Luo et al. (2017); Yao et al. (2020); Zhong et al. (2019)
4	Baicalin	C_21_H_18_O_11_	446.36	No	*Scutellaria baicalensis*; *Oroxylum indicum*; *Plantago major*	Shen et al. (2019); Zhang et al. (2017); Zhu et al. (2020a); Zhu et al. (2020b)
5	Chrysin	C_15_H_10_O_4_	254.24	No	*Oroxylum indicum*; *Passiflora caerulea*	Dou et al. (2013); Garg and Chaturvedi (2022)
6	Diosmetin	C_16_H_12_O_6_	300.26	No	*Citrus limon*; *Citrus medica*; *Mentha spicata*; *Mentha canadensis*	Li et al. (2021d); Yu and Liu (2021)
7	Diosmin	C_28_H_32_O_15_	608.54	Mild nervous disorder	*Citrus limon*; *Citrus medica*; *Mentha spicata*; *Mentha canadensis*	Gerges et al. (2022); Shalkami et al. (2018)
8	Eupatilin	C_18_H_16_O_7_	344.32	No	*Artemisia argyi*; *Artemisia asiatica*; *Artemisia princeps*	Sapkota et al. (2017); Zhou et al. (2018)
9	Fortunellin	C_28_H_32_O_14_	592.55	Not clear	*Citrus japonica*	Xiong et al. (2018)
10	Licoflavone B	C_25_H_26_O_4_	390.47	Not clear	*Glycyrrhiza uralensis*; *Glycyrrhiza inflata*; *Glycyrrhiza glabra*	Zhang et al. (2022)
11	Linarin	C_28_H_32_O_14_	592.55	No	*Buddleja officinalis*; *Mentha arvensis*; *Mentha haplocalyx*; *Chrysanthemum indicum*	Jin et al. (2022); Mottaghipisheh et al. (2021)
12	Lonicerin	C_27_H_30_O_15_	594.52	Not clear	*Lonicera japonica*	Lv et al. (2021)
13	Luteolin	C_15_H_10_O_6_	286.24	No	*Lonicera japonica*; *Chrysanthemum indicum*; *Perilla frutescens*	Li et al. (2016); Li et al. (2021a); Li et al. (2021b); Suga et al. (2021); Vukelic et al. (2020); Zuo et al. (2021)
14	Nobiletin	C_21_H_22_O_8_	402.39	No	*Citrus sinensis*; *Citrus paradise*; *Citrus aurantum*	Hagenlocher et al. (2019); Xiong et al. (2015)
15	Oroxylin A	C_16_H_12_O_5_	284.26	No	*Oroxylum indicum*; *Scutellaria baicalensis*; *Scutellaria rehderiana*	Sajeev et al. (2022a); Zhou et al. (2017)
16	Pectolinarigenin	C_17_H_14_O_6_	314.29	No	*Cirsium chanroenicum*; *Cirsium setidens*; *Cirsium japonicum*	Cheriet et al. (2020); Feng et al. (2022)
17	Sinensetin	C_20_H_20_O_7_	372.37	No	*Citrus sinensis*; *Citrus reticulata*; *Citrus aurantium*	Jie et al. (2021); Xiong et al. (2019)
18	Tangeretin	C_20_H_20_O_7_	372.37	No	*Citrus unshiu*; *Citrus reticulata*; *Citrus depressa*	Chen et al. (2021a); Eun et al. (2017)
19	Tricin	C_17_H_14_O_7_	330.29	No	*Medicago sativa*; *Triticum aestivum*; *Hordeum vulgare*	Li et al. (2021f); Shalini et al. (2016)
20	Wogonin	C_16_H_12_O_5_	284.26	Weak developmental toxicities and genotoxicities	*Scutellaria baicalensis*	Zhao et al. (2011); Zhou et al. (2022)
21	Wogonoside	C_22_H_20_O_11_	460.4	No	*Scutellaria baicalensis*	Huang et al. (2020); Liu et al. (2020b); Sun et al. (2015)

**TABLE 2:** Molecular mechanisms of naturally derived flavones against ulcerative colitis.

Name	Model	Effective dosage	Route of administration	Mechanism of action	References
Acacetin	DSS elicited UC in C57BL/6 mice	50, 150 mg/kg	Oral gavage	Up: *Firmicutes*; *Turicibacter* Down: TNF-α; IL-6; IL-1β; COX-2; iNOS; *Bacteroidaceae*; *Deferribacteres*; *Deferribacteraceae*; *Enterobacteriaceae*; *Escherichia-Shigella*; *Faecalibaculum*; *Proteobacteria*	Ren et al. (2021)
Apigenin	TNBS or DSS induced UC in Wistar rats or C57BL/6 mice	1, 3 and 10 mg/kg	Dietary administration	Down: MMP-3; TNF-α; IL-6; IL-1β; IL-18; COX-2; mPGES; iNOS; NALP3; Cl-caspase-1; pro-caspase-11; Cl-caspase-11	Marquez-Flores et al. (2016)
Baicalein	TNBS or DSS evoked UC in Balb/c mice or ICR mice or C57BL/6 mice	10, 20, 40 mg/kg *in vivo*	Oral administration (*in vivo*)	Up: ZO-1; occludin; IκBα; CYP1A1; CD4^+^ CD25^+^ Foxp3^+^ T cells; IL-10; IL-22; TGF-β; AhR (nuclear); RORγt^+^ IL-22 ILC3	Li et al. (2021g); Liu et al. (2020a); Luo et al. (2017); Yao et al. (2020); Zhong et al. (2019)
	LPS stimulated THP-1 cells and RAW264.7 cells	10, 25, 50 μM *in vitro*		Down: MPO; NO; iNOS; COX-2; ICAM-1; MCP-1; TNF-α; IL-1α; IL-1β; IL-6; IL-17; IL-17A; TLR4; MyD88; p-IκBα; p65; p-p65; p-p38; p-IRAK-1; p-JNK; p-ERK; NLRP3; ASC; caspase-1; MD-2/TLR4; Cyclin D1; STAT3; p-STAT3; IKKβ; CD11b^+^ cells; NOD2; SphK1; S1PR1; CD4^+^ IL-17^+^ T cells; AhR (cytosol)	
Baicalin	TNBS or DSS induced UC in SD rats or C57BL/6 mice	25, 50, 100, 120, 150 mg/kg *in vivo*	Oral gavage (*in vivo*)	Up: ZO-1; occludin; SOD; CAT; GSH; GSH-Px; IκB-α; IL-10; Bcl-2; FOXP3; β-catenin; CD4^+^CD25^+^ Foxp3^+^ cells; *Firmicutes*; *Bacteroidaceae*; *Christensenellaceae*; *Peptosreptococcaceae*; *Ruminococcaceae*; *Clostridiales*; acitric acid; butyric acid; propionic acid; *Butyricimonas* spp.; *Roseburia* spp., *Subdoligranulum* spp.; *Eubacteriu* spp.	Shen et al. (2019); Zhang et al. (2017); Zhu et al. (2020a); Zhu et al. (2020b)
	LPS stimulated RAW264.7 cells or HT-29 cells	5, 10, 20, 40 μM, 100 ng/mL *in vitro*		Down: MPO; NO; TNF-α; IL-1β; IL-6; IL-17; IL-33; p65; p-p65; p-IκBα; MDA; PGE_2_; caspase-3; caspase-9; Bax; Bcl-2/Bax; Cyt-c; p-IKKβ/IKKβ; p-IKBα/IKBα; RORγt; CD4^+^ IL17^+^ cells; Th17/Treg; p-PI3K/PI3K; p-AKT/AKT; Fas; FasL; *Proteobacteria*; *Actinobacteria*	
Chrysin	DSS evoked UC in C57BL/6 mice	25 mg/kg	Oral gavage	Up: IκBα; Cyp3a11; MDR1	Dou et al. (2013)
				Down: p-p65; p-IκBα; iNOS; COX-2; ICAM-1; TNF-α; MCP-1; IL-6; MPO	
Diosmetin	TNBS evoked UC in Wistar rats	25, 50, 100, and 200 mg/kg *in vivo*	Oral gavage (*in vivo*)	Up: ZO-1; occludin; claudin-1; TEER; SOD; CAT; GSH-Px; GSH; circ-Sirt1; Sirt1; total Nrf2; nuclear Nrf2; HO-1; *Bacteroidetes*; *Cyanobacteria*; *Odoribacteraceae*; *Prevotella*; *Rikenellaceae*; *Ruminococcus*; *Coprococcus*; *Roseburia*; *Oscillospira*, *Anaeroplasma*; *Synergistales*	Li et al. (2021d); Yu and Liu (2021)
	DSS evoked UC in C57BL/6 mice	25, 50, and 100 μM *in vitro*		Down: MDA; TNF-α; IL-1β; IL-6; IFN-γ; NF-κB; acetyl-NF-κB; apoptotic index; COX-2; *Firmicutes*; *Eggerthella*; *Flavobacterium*; *Clostridium*	
	LPS stimulated Caco-2 and IEC-6 cells				
Diosmin	TNBS induced UC in Wistar rats	5, 10, 25, and 50 mg/kg	Oral administration	Up: GSH	Shalkami et al. (2018)
	AA induced UC in Swiss albino rats			Down: TNF-α; COX-2; MPO; MDA; caspase-3	
Eupatilin	DSS evoked UC in C57BL/6 mice	10, 20 mg/kg *in vivo*	Oral administration (*in vivo*)	Up: ZO-1; occludin; IκB; p-AMPK/AMPK	Zhou et al. (2018)
	LPS induced THP-M macrophage TNF-α damaged NCM460 cells	5 and 10 μM *in vitro*		Down: TNF-α; IL-1β; MPO; ROS; NOX4; p-ERK/ERK; p-pJNK/pJNK; p-p38/p38; p-p65/p65	
Fortunellin	TNBS elicited UC in SD rats	20 and 80 mg/kg	Gavage administration	Up: SOD; GSH; Bcl-2; p-Akt; miR-374a; TEER	Xiong et al. (2018)
				Down: IL-6; TNF-α; IL-1β; MPO; MDA; ROS; PTEN; Cl-caspase-3; Bax; p-GSK-3β	
Licoflavone B	DSS elicited UC in C57BL/6 mice	40, 80 and 120 mg/kg	Oral administration	Up: ZO-1; occludin; claudin-1; IL-10; *Bacteroidetes*; *Lactobacillus*; *Ileibacterium*; *Enterobacter*; *Vibrionaceae*; *Faecalibacterium*; *Tannerellaceae*; *Adlercreutzia*; *Ahniella*; *WX65*; *Faecalibaculum*; *Rhodanobacteraceae*; *Streptococcus*; *Micromonospora*; *Bacteroides*; *Eubacterium_coprostanoligenes*; *Parabacteroides*	Zhang et al. (2022)
				Down: IL-4; IL-6; TNF-α; IL-1β; p-ERK; p-p38; p-JNK; p-ERK/ERK; *Firmicutes*; *Firmicutes*/*Bacteroidetes*; *Enterococcus*; *Alloprevotella*; *Eubacterium_ruminantium_group*	
Linarin	DSS evoked UC in C57BL/6J mice	25 and 50 mg/kg	Oral gavage	Up: mucin 2; ZO-1; occludin; claudin-1; IL-10; acetic acid; butyric acid; propionic acid; isobutyric acid; isovaleric acid; valeric acid	Jin et al. (2022)
				Down: MPO; IL-6; IFN-γ; TNF-α; IL-1β	
Lonicerin	DSS evoked UC in C57BL/6 mice	3, 10, and 30 mg/kg *in vivo*	Oral administration (*in vivo*)	Up: ATG5; NF-κB p65 (cytosol); LC3B-I/II	Lv et al. (2021)
	LPS stimulated THP-1 or BMDMs cells	1, 3, 10, and 30 μM *in vitro*		Down: MPO; F4/80^+^ macrophages; Cl-caspase-1; IL-1β; IL-18; IL-6; TNF; IL-1; NLRP3; EZH2; NF-κB p65 (nuclear); p-p65; p62	
Luteolin	DSS elicited UC in C57BL/6 mice or Wistar rat	5, 10, 20, 50 and 100 mg/kg *in vivo*	Oral administration (*in vivo*)	Up: ZO-1; occludin; claudin 1; TEER; SOD; CAT; Nrf2; HO-1; NQO1; p-ERK1/2; proliferating cells/crypt; SHP-1; PPAR-γ; *Bacteroidetes*; *Bacteroidaceae*; *Lactobacillus*; *Lachnospiraceae_NK4A136_group*; *Roseburia*; *Bacteroides*; *Butyricicoccus*; arginine metabolism; proline metabolism; starch metabolism; sucrose metabolism	Li et al. (2016); Li et al. (2021a); Li et al. (2021b); Suga et al. (2021); Vukelic et al. (2020); Zuo et al. (2021)
	TNF-α, and IFN-γ stimulated Caco-2 cells 5-HT stimulated RBL-2H3 cells	25, 50, 100, and 150 μM *in vitro*		Down: MDA; TNF-α; IL-1β; IL-6; p-JNK1/2; p-p38; p-Akt; p-STAT3; NF-κB p65; TNF-α; COX-2; Cl-caspase-3; caspase-9; Cl-caspase-9; cleaved PARP; p21; LC3B-II; Atg5; HMGB1; TLR4; MyD88; p-p65/p65; TPH-1; 5-HT; CLDN2; p-STAT3; STAT3; IL-17; IL-23; *Firmicutes*; *Proteobacteria*; *Prevotella_9*; the ability of DNA repair; ribosome metabolism; purine metabolism	
Nobiletin	TNBS elicited UC in SD rats	20, 40 and 50 mg/kg *in vivo* 10, 20, 40 and 80 μM *in vitro*	Oral administration (*in vivo*)	Down: TEER; MPO; mast cells; TNF-α; IL-1β; IL-6; NO; PGE_2_; iNOS; COX-2; MLCK; p65; PI3K; p-Akt; CCL2; COL13A1	Hagenlocher et al. (2019); Xiong et al. (2015)
	IL-10^−/−^ BALB/c mice				
	LPS stimulated Caco-2 cells				
	LPS stimulated human intestinal fibroblasts				
Oroxylin A	DSS evoked UC in C57BL/6 mice	100 and 200 mg/kg *in vivo*	Dietary administration (*in vivo*)	Up: p65 (cytoplasm), IκBα	Zhou et al. (2017)
	LPS combined ATP stimulated THP-Ms or BMDMs cells	25, 50 and 100 μM *in vitro*		Down: MPO; iNOS; TNF-α; IL-6; IL-1β; NLRP3; F4/80; p65; p-IκBα; Cl-IL-1β; Cl-caspase-1; ASC speck formation	
Pectolinarigenin	DSS elicited UC in C57BL/6J mice	2.5, 5 and 10 mg/kg *in vivo*	Oral administration (*in vivo*)	Up: Nrf2; IκBα; SOD; CAT; GSH; mucin 2	Feng et al. (2022)
	LPS stimulated RAW 264.7 and THP1 cell lines	10 and 20 μM *in vitro*		Down: NF-κB p-p65; p-IκBα; COX-2; iNOS; IL-6; IL-1β; TNF-α; MPO; MDA	
Sinensetin	TNBS evoked UC in SD rats	20 and 80 mg/kg *in vivo*	Oral gavage (*in vivo*)	Up: TEER; LC3Ⅱ/Ⅰ; p-ULK1; AMPK; p-AMPK	Xiong et al. (2019)
	TNF-α stimulated Caco-2 cells	20 and 40 μM *in vitro*		Down: IL-1β; IL-6; IFN-γ; MPO; p62; claudin-2; Cl-capsase-3; Cl-capsase-9	
Tangeretin	TNBS evoked UC in C57BL/6 mice	10 or 20 mg/kg *in vivo*	Oral administration (*in vivo*)	Up: ZO-1; occludin; claudin-1; IL-10; Foxp3; Tregs differentiation; *Firmicutes*; *Lachnospiraceae*; *Lactobacillaceae*; valeric; acetic acids; butyric acids	Chen et al. (2021a); Eun et al. (2017)
	LPS stimulated DCs	5, 10, and 20 μM *in vitro*		Down: TNF-α; IL-1β; IL-12; IL-17; IL-23; IL-12/IL-10; TNF-α/IL-10; IFN-γ; iNOS; COX-2; MPO; differentiation of Th1 and Th17 cells; MHC Ⅱ; CD40; CD80; CD86; p-p65; T-bet; RORγt; p-TAK1; p-IκBα; p-IRAK1; p-IKK-α/β; p-p38; p-ERK; p-JNK; *Bacteroidetes*; *Rikenellaceae*; *Marinifilaceae*; *Enterobacteriaceae*; *Rikenellaceae_RC9_gut_group*; *Alistipes*	
Tricin	DSS evoked UC in BALB/c mice	75, 100 and 150 mg/kg *in vivo*	Oral gavage (*in vivo*)	Up: *Bacteroidetes*	Li et al. (2021f)
	LPS stimulated RAW264.7 cells	12.5, 25 and 50, μM *in vitro*		Down: Nitrite; TNF-α; IL-1β; IL-6; MIP-2; MPO; MDSC; Treg cells; p-p65; *Firmicutes*; *Proteobacteria*; *Deferribacteres*; *Helicobacter*; *Ruminiclostridium_5*; *Streptococcus*; *Veillonella*; *Mucispirillum*; *Klebsiella*; *Haemophilus*	
Wogonin	DSS induced UC in BALB/c mice	30 mg/kg	Oral gavage	Up: SOD; GST; GSH; IL-10; Bax; caspas-3; caspas-9; Nrf2; HO-1	Zhou et al. (2022)
				Down: MPO; NO; TBARS; TNF-α; IL-6; PGE_2_; Bcl-2; COX-2; iNOS; TLR4; p-p65	
Wogonoside	DSS evoked UC in Balb/C or C57BL/6 mice	12.5, 25 and 50 mg/kg *in vivo*	Oral gavage (*in vivo*)	Up: ZO-1; occludin; claudin1; TEER; NF-κB (cytoplasm); TXNIP	Huang et al. (2020); Liu et al. (2020b); Sun et al. (2015)
	LPS stimulated THP-1 cells TNF-α stimulated Caco-2 cells	12.5, 25 and 50 μM *in vitro*		Down: MPO; iNOS; TNF-α; IL-1β; IL-6; IL-13; IL-18; IFN-γ; MIP-1α; NF-κB (nucleus); p-IκBα; p-p65; Cl-caspase-1; pro-caspase-1; Cl-caspase-1/pro-caspase-1; Cl-IL-1β; NLRP3; ASC; CD11b^+^F4/80^+^ monocyte/macrophages; CD11b^+^Gr-1^+^ neutrophils; F4/80^+^ cells; pMLC2/MLC2; MLCK	

Acacetin is a natural product discovered in many plants, including *Acacia farnesiana*, *Saussurea involucrata*, and *Turnera diffusa* (Wu et al., 2018). Researchers reported that acacetin possesses multiple pharmacologic functions covering anti-cancer, anti-inflammation, anti-obesity, cardioprotection, and neuroprotection (Singh et al., 2020). Ren et al. (2021) probed whether acacetin could improve UC in mice induced by DSS. Results showed that acacetin alleviated the clinic signs of DSS-treated UC, as determined by weight reduction, diarrhoea, colonic shortening, inflammatory infiltration, and histologic damage. Acacetin was found to suppress the *in vitro* inflammatory response of macrophages as well as the production of inflammation mediators in UC murine models. Moreover, a few characters of the intestinal microflora were disordered in DSS-treated UC mice, as manifested by a prominent decrease in microflora richness and a remarkable alteration in bacteria profiles. Whereas, acacetin administration inhibited this unbalance and recovered intestine microflora to levels consistent with normal group. Collectively, the results manifested that acacetin could mitigate DSS-treated UC in mice, at least in part, by suppressing inflammation and modulating the gut microflora.

Apigenin is a usual dietary flavone which is broadly existent in some fruits, vegetables and pharmaceutical plants. It has various bioactivities involving anti-cancer, anti-oxidation, anti-inflammation, anti-bacteria, and anti-virus (Ginwala et al., 2019; Salehi et al., 2019). Marquez-Flores et al. (2016) elucidated the protective effect and mechanism of dietary apigenin enrichment in DSS-evoked colitis murine models. Apigenin supplementation reduced the macroscopic symptoms and histopathological injury of UC, according to the findings. Moreover, it reduced the expression of mPGES, COX-2 and iNOS in colon tissues and decreased the serum MMP-3 level. Likewise, apigenin diet decreased TNF-α and IL-1β secretion in LPS-activated splenocytes. In addition, apigenin’s anti-inflammatory effect was linked to the suppression of both canonical and non-canonical NLRP3 inflammasome paths via modulating cleaved caspase-1 and caspase-11 enzymes to reduce IL-1β and IL-18 expressions. In conclusion, an apigenin supplement may offer a basis for formulating a novel dietary method for preventing and treating UC.

Baicalein, a main active constituent in the roots of *Scutellaria baicalensis*, was testified to possess multifarious effects involving anti-cancer, anti-inflammation, anti-oxidation, anti-hepatotoxicity, as well as neuroprotection (Dinda et al., 2017; Song et al., 2021). Luo et al. (2017) probed the function and mechanism of baicalein against UC in TNBS-evoked UC model. Experimental results revealed that baicalein relieved the seriousness of TNBS-treated UC in mice through reducing MPO activity and pro-infammatory factors expression. The downregulation of NF-κB and p38 MAPK was related to the reduced expression of TLR4 and its adaptor MyD88 in mucosa. *In vitro*, baicalein suppressed the TLR4/MyD88 signal cascades (NF-κB and MAPKs) in LPS-activated macrophages. Besides, baicalein could bind to the hydrophobic domain of the MD-2 pocket and restrain the formation of LPS-evoked MD-2/TLR4 complex. Moreover, baicalein decreased NLRP3 infammasome excitation and downriver IL-1β expression in a dosage-dependent mode. Therefore, these results indicated that baicalein might ameliorate TNBS-treated UC by the suppression of TLR4/MyD88 signal cascade and desactivation of NLRP3 infammasome. Zhong et al. (2019) found that the protection of baicalein on UC was correlated with inhibiting NF-κB and STAT3 signal pathways. Yao et al. (2020) discovered that baicalein administration alleviated UC in mice via suppressing S1P-STAT3 signal pathway. In the experiment of Liu C. et al. (2020), they proved that baicalein could prevent DSS-evoked UC, and the therapeutical mechanism may be relevant with the modulation of Th17/Treg differentiation by AhR excitation. In another research, Li Y. Y. et al. (2021) demonstrated that baicalein ameliorated UC through improving the enteric epithelia barrier by AhR/IL-22 pathway in ILC3s. Taken together, baicalein may be beneficial in the therapy of UC.

Baicalin is a biologically active flavone glycoside separated from the dry roots of *S. baicalensis* and possesses diversified effects covering anti-virus, anti-inflammation, anti-bacteria, hepatoprotection, and cardioprotection (Guo et al., 2019; Jin et al., 2019). The role and mechanism of baicalin in the treatment of colitis was investigated by Zhu et al. (2020a). Experimental data showed that baicalin observably alleviated TNBS-elicited UC through decreasing the levels of pro-inflammatory factor (TNF-α, IL-6 and IL-1β), elevating the content of anti-inflammatory mediator IL-10, and enhancing the expression of TJ proteins ZO-1 and β-catenin, which may be attained by blocking the PI3K/AKT signal path. Furthermore, baicalin obviously restrained the disequilibrium between pro- and anti-inflammatory cytokines and markedly reduced apoptosis through blocking the PI3K/AKT signal path in LPS-treated HT-29 cells, which was consistently in line with the *in vivo* finding. Therefore, these results illustrated that baicalin could treat colitis by the inhibition of PI3K/AKT signal pathway. Zhu et al. (2020b) further found that baicalin probably protected mice against UC via maintaining Th17/Treg balance and modulating both gut microflora and SCFAs. Among the research from Zhang et al. (2017), they proved that baicalin could attenuate DSS-evoked UC via restraining IL-33 expression and NF-κB excitation. Shen et al. (2019) also discovered that baicalin exerted a regulatory role on the IKK/IKB/NF-κB signal cascade and apoptosis-associated proteins in murine models of colitis. Collectively, baicalin might be a perspective therapy candidate for colitis.

Chrysin, a natural flavone in many plants, including *Oroxylum indicum* and *Passiflora caerulea*. Studies proved that chrysin has anticancerous, antidiabetic, antidepressive, immunoregulatory, and neuroprotective activities (Kasala et al., 2015; Garg and Chaturvedi, 2022). Dou et al. (2013) evaluated the efficacy of chrysin as a putative murine PXR activator in suppressing UC. Chrysin administration alleviated inflammatory signs in DSS or TNBS induced murine models and caused a decrease in NF-κB target genes (e.g., iNOS, COX-2, ICAM-1, and MCP-1) in the colonic tissues. Chrysin restrained the phosphorylation/degradation of IκBα, resulting in lower colonic of MPO, TNF-α and IL-6 levels. Consistent with the *in vivo* findings, chrysin prevented LPS-activated migration of NF-κB p65 into the nucleus of RAW264.7 cells. Moreover, chrysin dosage-dependently stimulated human/murinse PXR in reporter gene experiments and up-modulated xenobiotic detoxification genes in the colonic mucosa, but not in the liver. RNA interference-mediated silencing of PXR demonstrated the crucial role that PXR plays in chrysin’s induction of xenobiotic detoxification genes and excitation of NF-κB. *In vitro* PXR transduction experiments confirmed that chrysin suppresses NF-κB transcriptional activity. The results suggested that chrysin’s role in UC inhibition is primarily mediated by the PXR/NF-κB pathway. In conclusion, chrysin has the potential to be developed as gut-specific PXR agonists.

Diosmetin, a flavone commonly found in citrus plants, exhibits a wide range of effects covering anti-inflammation, anti-tumor, anti-apoptosis, and anti-bacteria (Zhang et al., 2012; Patel et al., 2013). A study conducted by Yu and Liu (2021) investigated the mechanism and efficacy of diosmetin in treating UC using TNBS-induced colitis rats as a model. Experimental results exhibited that diosmetin notably reduced colon anabrosis and inflammatory symptoms in the colon mucosa. TNBS-induced decrease of SOD and CAT was observably inhibited, but MDA content in the colon tissues was reduced. After diosmetin administration, the concentrations of TNF-α, IL-6 and NF-κB and the quantity of apoptotic cells were markedly decreased. In another research, Li H. L. et al. (2021) investigated the function and mechanism of diosmetin against UC in DSS-evoked UC mice. Results demonstrated that diosmetin exerted therapeutical roles in DSS-treated UC by several paths, involving the decrease of proinflammatory cytokines and oxidant stress, the elevated expression of TJ proteins (ZO-1, claudin-1 and occludin), and the regulation of intestinal microflora. The circ-Sirt1/Sirt1 axis partially mediates the therapeutic effects of diosmetin on UC. However, further studies are needed to determine whether diosmetin modulates the composition of intestinal microflora through the Sirt1 pathway. Therefore, diosmetin could be effectively adopted to prevent and treat UC.

Diosmin is a dietary flavone glycoside abundantly existed in citrus plants. Numerous studies have proven that diosmin possesses anti-inflammatory, antioxidative, anticancerous, hepatoprotective, neuroprotective, and renoprotective effects (Zheng et al., 2020; Gerges et al., 2022). Shalkami et al. (2018) investigated the efficacy of diosmin on colitis. Evidences manifested that acetic acid led to a rise of DAI and colonic injury index scores. The indexes of inflammation (MPO, TNF-α and COX-2) and oxidant stress (MDA and decreased GSH) were notably increased. These variations were related to raise in colonic caspase-3 expression. Diosmin treatment dosage-dependently decreased the DAI and colonic injury index scores. Moreover, diosmin caused a prominent decline of inflammatory and oxidant stress markers in addition to decreasing caspase-3 level. Collectively, diosmin treatment reduced colitis progression, deciding by its capacity to suppress inflammation, oxidant stress, and apoptosis in the colons of rats.

Eupatilin is a natural flavone mainly existed in Artemisia plants, such as *Artemisia argyi*, *Artemisia asiatica* and *Artemisia princeps* (Nageen et al., 2020). Numerous studies indicated that eupatilin possesses multiple bioactivities ranging from anti-ulcer, anti-inflammation, and anti-cancer (Cho et al., 2011; Sapkota et al., 2017). Zhou et al. (2018) investigated the activity of eupatilin against UC and illustrated the mechanism. Experimental results elucidated that eupatilin significantly mitigated inflammatory reactions in LPS-provoked macrophages. Eupatilin notably safeguarded colon epithelia through reducing over expression of TJs and NOX4, and improving AMPK excitation in TNF-α excited NCM460 cells. Moreover, *in vivo* research proved that eupatilin therapy markedly alleviated the symptoms and pathological variations of UC murines. Administration of an AMPK pharmacologic suppressant in mice resulted in a decrease in the therapeutic effects of eupatilin. In brief, eupatilin could ameliorate DSS-treated murine colitis via restraining the inflammatory response and keeping the integrality of the intestine epithelia barrier by AMPK excitation.

Fortunellin, a natural dietary flavone mainly existed in *Citrus japonica*, has multifarious bioactivities covering anti-tumor, anti-oxidation, and anti-inflammation (Zhao et al., 2017; Panagiotopoulos et al., 2021). Xiong et al. (2018) explored the function and mechanism of fortunellin in treating UC using TNBS-induced colitis rats as a model. Fortunellin alleviated the clinical signs of UC, involving excessive inflammatory symptoms and oxidative stress. Fortunellin reduced the apoptosis of epithelial cells in UC by suppressing PTEN expression. Fortunellin-caused decrease of PTEN could be neutralized by miR-374a decline. Furthermore, miR-374a knockdown *in vivo* partially restrained the effect of fortunellin on UC model. Together, PTEN suppression facilitates the reinforced effect of fortunellin on UC. Fortunellin targeting miR-374a is a negative modulator of PTEN. This research offers new ideas into the pathologic mechanism and therapy alternatives of UC.

Licoflavone B is a minor flavone in licorice, which is a kind of medicinal and edible Chinese herbal medicine (de Carvalho et al., 2015; Liu et al., 2022). Zhang et al. (2022) examined the efficacy and mechanism of licoflavone B against UC in DSS-exposed C57BL/6 murines. Experimental result showed that licoflavone B notably restrained DSS induced weight reduction, DAI rise, histologic injury, and colon inflammation, manifesting that licoflavone B possesses a beneficial effect on UC. It was discovered that licoflavone B maintained the integrality of the colon barrier through suppressing the apoptosis of colon cells and increasing the levels of ZO-1, occludin, and claudin-1. Furthermore, licoflavone B remodeled the microbiota composition via restraining detrimental bacterium and promoting beneficial microbes. Besides, licoflavone B exhibited an anti-colitis effect via the blockage of the MAPK path. In conclusion, the results provided worthy information for the exploration of new anti-colitis drugs.

Linarin, a naturalflavone existed in the plants of Buddleja, Mentha, and Cirsium, possesses various activities covering anti-inflammation, anti-allergen, as well as hepatoprotection (Han et al., 2018; Mottaghipisheh et al., 2021). Jin et al. (2022) evaluated the protection of linarin against DSS-evoked colitis in C57BL/6J murines and explored the possible mechanisms. Experimental results displayed that linarin administration relieved the DSS-evoked histopathologic injury, and strengthened the mucosa layer and enteric barrier function. Significantly, linarin markedly lowered MPO vitality and the levels of pro-inflammatory factors, whereas increased the mRNA expression of anti-inflammatory factor in colonal tissues. Furthermore, linarin recovered the intestinal flora injured by DSS. Linarin also partially enhanced the relative amounts of SCFAs-generating bacterium and the levels of SCFAs. The findings of the study suggest that linarin may be a promising nutritional intervention for the treatment of UC.

Lonicerin is a flavone glycoside extracted from *Lonicera japonica*, the flower buds of which are often employed for treating inflammatory and infectious disorders (Xu et al., 2019). Lv et al. (2021) reported the therapeutical role of lonicerin on intestine inflammation via binding directly to EZH2 histone methyltransferase. The modification of H3K27me3 by EZH2 was found to promote ATG5 expression, which in turn results in elevatory autophagy and expedites autolysosome-mediated degradation of NLRP3. The dynamic simulation study shows that the mutation of EZH2 residues (His1 and Arg685) greatly reduces the protective role of lonicerin. Moreover, *in vivo* researches verified that lonicerin treatment disturbs the assembly of NLRP3-ASC-pro-caspase-1 complex and relieves UC in a dose-dependent manner, an effect that is attenuated via administering an EZH2-overexpressing plasmid. Therefore, the results suggest that lonicerin may be a potential anti-inflammatory epigenetic agent, and the EZH2/ATG5/NLRP3 axis could be a novel therapeutic target for the treatment of UC and other inflammatory ailments.

Luteolin is a common dietary flavone in some edible plants, including *Chrysanthemum indicum*, *L. japonica*, and *Perilla frutescens* (Ganai et al., 2021). Previous studies have demonstrated that possess numerous beneficial effects, including anti-carcinogenic, anti-oxidative, anti-inflammatory, anti-allergic, and antimicrobial effects (Nabavi et al., 2015; Aziz et al., 2018). Li et al. (2016) probed the function and mechanism of luteolin against UC in DSS-evoked colitis murines. The results displayed that luteolin prominently decreased DAI scores, and restrained colonal shortening and histologic injury. Furthermore, luteolin effectually lowered the levels of inflammatory factors, covering iNOS, TNF-α and IL-6. Administration of luteolin was found to increase the levels of colon contents of SOD and CAT, as well as the expression of Nrf2 and its downstream targets, such as HO-1 and NQO1. These findings suggested that luteolin may restrain UC by activation of the Nrf2 signal path. Among the study from Vukelic et al. (2020), the researchers discovered that luteolin has the potential to attenuate experimental UC by exerting anti-inflammatory, anti-apoptotic, and anti-autophagic effects. These effects are mediated by the inhibition of JNK1/2, p38, PI3K/Akt, NF-κB, and STAT3 signal paths, along with the induction of ERK1/2. In the research of Suga et al. (2021), they discovered that luteolin may possess the potential to relieve inflammation responses through reducing excessive 5-HT by inhibition of TPH-1 in RBL-2H3 cells. Zuo et al. (2021) proved that luteolin notably mitigated DSS-elicited UC, and the mechanism was associated with the modulation of enteric HMGB1-TLR-NF-κB signal path. In two other studies, Li et al. (2021a), Li et al. (2021b) indicated that luteolin could maintain intestine epithelia barrier function by suppressing STAT3 signaling and alleviate inflammatory reactions by regulating intestinal microflora in the treatment of colitis. Altogether, luteolin may be a prospective therapeutic drug for UC.

Nobiletin is a natural dietary flavone prevailingly found in a few citrus plants composed of *citrus sinensis*, *citrus paradise*, and *citrus aurantum* (Ashrafizadeh et al., 2020). Modern studies proved that nobiletin possesses multiple pharmacological actions, including anti-cancer, anti-inflammation, anti-oxidation, anti-dementia, and neuroprotection (Braidy et al., 2017). Hagenlocher et al. (2019) explored the function of nobiletin on inflammation and fibrosis in IL-10^−/−^ colitis. The results showed that nobiletin administration caused a decline of clinic signs and a longer life expectancy. Moreover, histologic scores of UC were decreased in comparation with normal groups. Administration of nobiletin in IL-10^−/−^ mice was found to decrease the number of mast cells and reduce their degranulation, which was positively correlated with the DAI. Besides, nobiletin administration also led to a decrease in fibrotic marker collagen deposition. In LPS stimulated human intestinal fibroblasts, the levels of collagen and pro-inflammatory factors COL13A1, IL-6, TNF, and CCL2 was down-modulated after nobiletin administration. Therefore, nobiletin administration was found to reduce signs and markers of inflammation, as well as the deposition and expression of fibrotic collagen in UC murines. Among another test, Xiong et al. (2015) investigated the effects of nobiletin on the exaggerated inflammatory reaction and weakened barrier function in UC rats. Experimental data manifested that nobiletin played anti-inflammatory roles in TNBS-treated UC by reducing iNOS and COX-2 levels. Nobiletin recovered the barrier function destroyed following TNBS treatment via the control of the Akt-NF-κB-MLCK signalling. In conclusion, nobiletin might be a prospective candidate for the treatment of colitis.

Oroxylin A, a main ingredient in the roots of *O. indicum* and *S. baicalensis*, possessed a wide range of beneficial bioactivities, including anti-inflammatory, antineoplastic, anticoagulative, cytoprotective, and neuroprotective bioactivities (Sajeev et al., 2022a; Sajeev et al., 2022b). Zhou et al. (2017) investigated the role of oroxylin A on DSS-treated mice UC by targeting NLRP3 inflammasome. The results displayed that oroxylin A alleviated UC, characterized by inhibiting weight reduction, colonic length shortening and inflammatory infiltration. The levels of TNF-α, IL-1β, and IL-6 in colons were also notably lowered by oroxylin A. Moreover, oroxylin A prominently inhibited the NLRP3 level in gut mucosa tissues. Besides, NLRP3 gene knockout mice had an obvious protective effect on UC treated by DSS, and oroxylin A administration exhibited no roles on relieving inflammatory symptoms in NLRP3^−/−^ murine. Further research discovered that oroxylin A dosage-dependently suppressed the excitation of NLRP3 inflammasome in BMDMs and THP-Ms, leading to a decrease in cleaved caspase-1 and cleaved IL-1β. Moreover, luteolin A mitigated the expression of NLRP3 protein depending on the suppression of p65 and nuclear transsituation. In addition, oroxylin A specifically inhibited the formation of ASC specks and the assembly of inflammasomes, both of which contributed to the blockade of the NLRP3 inflammasome. The above evidences indicated that oroxylin A suppressed NLRP3 inflammasome excitation and might be potentially employed to treat colitis.

Pectolinarigenin, a natural flavone existed in Cirsium and Citrus species, possesses different bioactivities including anti-inflammatory, antidiabetic, as well as anticancer properties (Lee et al., 2018; Cheriet et al., 2020). Feng et al. (2022) probed the possible protective effects of pectolinarigenin on LPS-treated macrophage cells and DSS-evoked UC mice. The results displayed that pectolinarigenin suppressed the LPS-evoked NF-κB excitation through disturbing IκB-α degradation. Subsequently, elevated Nrf2 protein expression was found on pectolinarigenin administrated RAW 264.7 and THP1 cells. Besides, they uncovered that pectolinarigenin mediated the NF-κB/Nrf2 path modulation, subsequently restrained the levels of iNOS, COX-2, IL-6, IL-1β, and TNF-α in RAW 264.7 and THP1 cells. Moreover, pectolinarigenin dosage-dependently mitigated colonic inflammation through adjusting NF-κB/Nrf2 signal path and improving MPO vitality and redox modulators in DSS-evoked UC mice. Likewise, we found the minimal pathologic injuries in the pectolinarigenin-treated mice colons, and the rise of goblet cell population and mucin-2 generation. Collectively, the results manifested that pectolinarigenin alleviated the DSS-evoked UC in murine through modulating the NF-κB/Nrf2 path. Therefore, pectolinarigenin may hold potential as a therapeutic agent for the treatment of UC.

Sinensetin, a dietary flavone mainly existed in citrus plants such as *Citrus sinensis*, was discovered to possess anticancerous, antioxidative, anti-inflammatory as well as antibacterial effects (Kim et al., 2020; Jie et al., 2021). Xiong et al. (2019) probed the therapeutic potential and underlying mechanism of sinensetin against UC in TNBS and DSS evoked UC rats. Sinensetin reversed colitis-related rise in intestine penetrability, notably facilitated epithelia cells autophagy, reduced epithelia cells apoptosis, and lowered mucosa claudin-2. Sinenstetin relieved the clinical signs in UC rats and mice. Knockdown of AMPK changed the encouragement of epithelia autophagy by sinensetin. Collectively, sinensetin markedly relieved intestine barrier dysfunction in UC through boosting epithelia cells autophagy, and further suppressing apoptosis and claudin-2 expression. Thus, these findings indicated the new promising benefit of sinensetin in UC.

Tangeretin is a polymethoxylated dietary flavone existed broadly in citrus plants, involving *Citrus unshiu*, *Citrus reticulata*, and *Citrus depressa*. Studies have proved that tangeretin possesses diversified biological activities, such as anti-inflammation, anti-cancer, and neuroprotection (Alhamad et al., 2021; Arafa et al., 2021). Eun et al. (2017) studied the therapeutic potential and underlying mechanism of tangeretin in treating UC. The results showed that tangeretin restrained TNF-α, IL-12, and IL-23 levels and NF-κB excitation in LPS-activated dendritic cells, but not affected IL-10 level. Moreover, tangeretin restrained the excitation and translocation of p65 into the nucleus *in vitro* through suppressing LPS binding to dendritic cells. Tangeretin treatment inhibited the inflammatory reactions, involving NF-κB and MAPK excitation and MPO vitality, in the colons of murine with TNBS evoked UC. Tangeretin elevated TNBS induced low expression of TJs including occludin, claudin-1, and ZO-1. Tangeretin also suppressed TNBS-evoked differentiation of Th1 and Th17 cells and the levels of T-bet, RORγt, interferon-γ, IL-12, IL-17, and TNF-α. Whereas, tangeretin elevated TNBS-restrained differentiation of regulatory T cells and the levels of Foxp3 and IL-10. The results suggested that tangeretin might ameliorate UC through repressing IL-12 and TNF-α productions and NF-κB excitation via the suppression of LPS bond on immunocytes. In another research of Chen B. et al. (2021), they also probed the therapeutic potential and underlying mechanism of tangeretin against UC in DSS-evoked colitis mice. Experimental results indicated that dietary tangeretin could relieve UC through restraining inflammatory reactions, recovering intestine barrier function, and regulating intestine microflora.

Tricin is a dietary flavone monomer broadly distributed in grains, and has antineoplastic, anti-inflammatory, and antiangiogenic properties (Shalini et al., 2016; Jiang et al., 2020). Li X. X. et al. (2021) studied the potential protective mechanism of tricin on LPS-stimulated RAW264.7 cells and probed the effect of tricin on UC mice treated by 4.5% DSS for 7 days. The result displayed that tricin observably decreased NO level in LPS stimulated RAW264.7 cells and the anti-inflammation role of tricin was proved to inhibit the NF-κB pathway. Moreover, tricin administration (150 mg/kg) markedly mitigated colonic length decline, decreased MPO vitality and DAI scores, and recovered the elevatory myeloid-derived suppressor cells in acute UC mice. The effect of DSS on intestinal flora, including the incremental population of *Proteobacteria* and *Ruminococcaceae*, was indicated to be alleviated by tricin therapy. Therefore, tricin could improve acute colitis through relieving colon inflammation and regulating intestinal microflora profile.

Wogonin, a natural flavone in *S. baicalensis*, has multifold functions, including anti-oxidation, anti-inflammation, anti-cancer, anti-virus, and neuroprotection (Wu et al., 2016; Huynh et al., 2020). Zhou et al. (2022) investigated the role and mechanism of wogonin against UC in DSS-treated colitis mice. Results exhibited that DSS strikingly reduced weight and colonic length, and elevated inflammatory symptoms in the colon. Wogonin effectively restrained colon ulcer, neutrophil infiltration and histologic variations elicited by DSS. The increase of pro-inflammatory factors (e.g., TNF-α, IL-6, COX-2, iNOS) and decreased activities of antioxidative enzymes (e.g., SOD, GST and GSH) were also notably regulated after wogonin treatment. In addition, wogonin activated apoptosis by suppressing Bcl-2 and elevating the contents of Bax, caspase-3, and -9. Further investigation revealed that wogonin’s protective role against DSS-induced UC was closely linked to the modulation of the Nrf2/TLR4/NF-B signaling.

Wogonoside, one of the main flavones from *S. baicalensis*, was discovered to possess multifarious pharmacologic actions including anti-inflammation, anti-cancer, and anti-oxidation (Chen et al., 2013; Liu Q. et al., 2020). Sun et al. (2015) assessed the action and mechanism of wogonoside against UC in DSS-elicited murine colitis. Experimental data certified that wogonoside dosage-dependently alleviated DSS-evoked weight reduction and colonic length shortening. Furthermore, wogonoside reversed DSS-evoked colon pathologic injury, notably restrained inflammation cell infiltration and reduced MPO and iNOS vitalities. The contents of pro-inflammatory mediators in serums and colons were also markedly suppressed by wogonoside. Besides, wogonoside significantly reduced the generation of IL-1β, TNF-α and IL-6 and restrained mRNA levels of pro-IL-1β and NLRP3 in PMA-differentiated monocytic THP-1 cells by preventing the activization of NF-κB and NLRP3 inflammasome. Therefore, these evidences verified that wogonoside exhibited an anti-inflammatory function via control of NF-κB and NLRP3 inflammasome. Among the experiment of Huang et al. (2020), the researchers explored whether wogonoside regulates intestine barrier function. The results manifested that wogonoside mitigated gut inflammation in UC and exerted a protective action on gut epithelia barrier function both *in vivo* and *in vitro*. Meanwhile, it was proven that wogonoside regulated gut epithelia TJs primarily via suppressing the MLCK/pMLC2 signal pathway. Additionally, Liu Q. et al. (2020) also explored the tissular distribution of wogonoside and its therapeutical effect on UC and the probable mechanism. Results testified that wogonoside could be absorbed by the colon and relieve inflammation reactions by suppressing NLRP3 inflammasome formation and excitation, which was associated with an inhibiting role on the TXNIP-dependent NF-κB signal pathway. In summary, wogonoside shows promise as a new medication for treating UC.

## Discussion

UC is a multifactorial persistent inflammatory bowel disorder. The destruction of the gut barrier is associated with UC and can result in pathogenetic antigen intrusion (Lee et al., 2020). Therefore, novel means of restoring the epithelial and mucus barrier, facilitating mucosa healing, and decreasing mucosa penetrability are regarded as promising methods for treating UC (Li J. J. et al., 2021). TJ forms a paracellular osmotic barrier to limit the traverse of ions, small solutes, and water (Han et al., 2023). Among various proteins that make up the TJ, ZO are the main tightly linked cytoskeletal proteins related to epithelia integrality, occludin is a crucial protein in maintaining barrier function and TJ stabilization, while claudin-1 is a membrane-spanning protein that forms part of the TJ strands (Atsugi et al., 2020; Kuo et al., 2021). The formation and disruption of the multiprotein complex made up of ZO-1, occludin and claudin-1 in intestinal epithelia cells could observably influence the intestine epithelia barrier function (Lan et al., 2021; Liu and Zhu, 2022). In the present review, natural flavones involving diosmetin, licoflavone B, luteolin, tangeretin, and wogonoside were found to notably reduce paracellular permeability and increase TEER value which was concomitant with increased levels of TJ proteins (ZO-1, occludin, and claudin-1). These evidences indicated that flavones could suppress the breakdown of intestinal barrier in UC models evoked by DSS, TNBS and LPS *in vivo* and *in vitro*.

Oxidant stress exerts a crucial action in pathophysiology of UC (Wojcik-Grzybek et al., 2022). Studies have revealed that increased production of ROS destroys the cellular macromolecules (DNA, lipids and proteins) and breaks epithelia cell integrality (Juan et al., 2021). Superoxide anion, continuously produced through endogenous processes and exogenous sources, is the main free radical to induce oxidative injury, which can be restrained by the first-line defense enzyme systems like SOD and CAT (Qiu et al., 2021). SOD converts the superoxide anion into H_2_O_2_, a metabolite that is easy to diffuse and stable, and then CAT further neutralizes H_2_O_2_ into water (Yu et al., 2023). CAT could enhance the oxidative resistibility and preserve the low steady state level of ROS. GSH, as a capital hydrophilic and intracellular nonenzymatic antioxidant, exerts an important effect in relieving tissue injury through eliminating reactive oxygen and nitrogen species (Ighodaro and Akinloye, 2018; Wu et al., 2021). Early studies showed that GSH concentration was found to be evidently decreased in colonic tissues when antioxidants were neutralized by released oxygen derived free radicals (Hu et al., 2021). The reduction of GSH can further cause a rise in MDA level, a final product of lipid peroxidation that finally brings about oxidative injury. As demonstrated in the present review, baicalin, diosmetin, fortunellin, luteolin, and wogonin could notably elevate the levels of the antioxidative enzymes SOD, GSH, GST, and CAT and suppress ROS and MDA activity induced by AA, DSS or TNBS treatment in the colitis tissue. These data indicated that natural flavones can effectively alleviate colitis by reducing oxidative stress.

Hyperactive inflammation cells, particularly neutrophils and macrophages, generate some pro-inflammatory factors, ROS, MPO and nitrogen metabolites, which are related to the pathogenic mechanism of colitis (Saraiva et al., 2022). Moreover, COX-2 and iNOS are pro-inflammatory enzymes that can be induced in inflamed tissues, leading to the generation of NO and PGE_2_ (Zhao et al., 2022). Elevated production of inflammatory cytokines, such as TNF-α, IL-1β, and IL-6, could injure gut epithelia cells and exacerbate colitis (Ahmad et al., 2021). IL-10 takes part in a Th2-mediated immunological response, and suppresses TNF-α, IL-1β, and IL-6 levels, exerting an anti-inflammatory action in the intestine mucosa immunity system. Thus, modulating the balance between pro- and anti-inflammatory factors is regarded as a necessary therapy approach for colitis (Yin et al., 2020; Zhuang et al., 2021). In this review, we firstly revealed that all natural-derived anti-colitis flavones could remarkably modulate the balance of pro- and anti-inflammatory cytokines in colonic tissues of colitis murine via attenuating pro-inflammatory agents and elevating anti-inflammatory factors.

The intrinsic immunity system identifies the existence of specific bacteria antigens through a broad family of pattern-recognition receptor (PRR). TLR4 belongs to the PRR that can recognize LPS (endotoxin), a main ingredient of gram-negative bacteria’s outer membrane, and stimulate the generation of pro-inflammatory factors, causing an inflammatory reaction (Dai et al., 2022). Moreover, TLR4 level was elevated in colon tissues and gut epithelia cells of UC animals and patients (Fajstova et al., 2020). Convincing proofs proves the therapeutic role of restraining TLR4/MyD88 signal molecules, subsequently resulting in the devitalization of NF-κB and MAPKs, and the suppression of pro-inflammatory factors (Ma et al., 2020). In this review, we found that natural-derived flavones, including baicalein, luteolin and wogonin observably restrained the enhancement of TLR4 and MyD88 in TNBS or DSS-treated UC murine and in LPS-treated macrophages or intestinal epithelial cells. Subsequently, the TNBS or LPS-treated excitation of NF-κB and MAPKs pathways and their downstream regulatory genes, including pro-inflammatory mediators (e.g., TNF-α, IL-6, and IL-1β), adhesion molecules (e.g., ICAM-1), NO and PGE_2_ were suppressed. These results showed that downregulation of TLR4/MyD88 signal cascades was associated with the anti-inflammatory roles of the above natural-derived anti-colitis flavones in UC models *in vivo* and *in vitro*.

Besides, the generation of pro-inflammatory factors is also modulated by a multiprotein complex known as inflammasome, which mainly consists of 3 ingredients including NLR, ASC and caspase-1. Among a variety of inflammasome, NLRP3 inflammasome composed of NLRP3, ASC and pro-caspase-1 is the most widely investigated (Mangan et al., 2018). The NLRP3 inflammasome plays a vital role in regulating the inflammatory response in the intestines and maintaining homeostasis by mediating multiple pro-inflammatory signals (Busch et al., 2022; Martinez-Lopez et al., 2022). After stimulation, NLRP3 recruits ASC adaptor to accelerate the recruitment of pro-caspase-1. Pro-caspase-1 then aggregates and automatically splits to produce active caspase-1. Activating caspase-1 is needed to transform pro-IL-1β and pro-IL-18 into their mature active forms IL-1β and IL-18 (Paik et al., 2021). Subsequently, IL-1β and IL-18 are secreted outside of the cell, trigger the “waterfall” cascade of downstream signaling, and participate in the development of numerous inflammatory illnesses, including UC (Chen C. et al., 2021). Chen et al. discovered that the serum levels of NLRP3, caspase-1, HMGB1 and IL-1β were significantly increased in UC patients (Chen et al., 2020). In congruence with previous investigations, in this review, we discovered that natural-derived anti-colitis flavones, such as baicalein, oroxylin A, lonicerin and wogonoside notably preserved IL-1β and IL-18 production, which was linked to the suppression of NLRP3, ASC and caspase-1 in a dosage-dependent mode both in TNBS- or DSS-induced UC murine and LPS-treated THP-1 or RAW264.7 cells.

Furthermore, research personnel obtained crucial insight into gut microflora constitution during gut inflammation and have uncovered novel directions for colitis therapy (Li et al., 2020). Some studies have emphasized that the prominent variations in intestinal microflora constitution in colitis sufferers can bring about intestine inflammation (Wang et al., 2022). Serious imbalance is primarily presented in the intestine of colitis sufferers, where there is a decrease in *Firmicutes* and *Bacteroidetes*, and an increase in *Proteobacteria*. The elevated endotoxin generated by *Proteobacteria* may cause damage to the gut’s penetrability, resulting in adhesion and invasion of gut epithelia cells, destruction of the host’s defenses, excitation of the inflammatory reaction, alterations in the composition of intestinal microflora, and eventually facilitating the occurrence of colitis (Jang et al., 2019). Moreover, *Enterobacteriaceae*, facultative anaerobic bacteria, are associated with the pathogenesis of colitis through aggravating intestinal inflammation and barrier injury (Kim et al., 2021; Qin et al., 2021). *Escherichia-Shigella*, gram-negative bacteria with an outer LPS membrane, can intrude into the colon epithelia and cause intestinal inflammation (Dai et al., 2021). *Roseburia* and *Lactobacillus* are probiotic strains with vital roles in maintaining intestinal homeostasis, inducing host’s colonic goblet cells, modulating immune system and promoting SCFAs productions (Zhang et al., 2020; Wang et al., 2021). In this review, the levels of *Proteobacteria*, *Enterobacteriaceae* and *Escherichia-Shigella* were reduced, while those of *Bacteroidetes*, *Roseburia* and *Lactobacillus* were elevated after acacetin, baicalin, diosmetin, luteolin, licoflavone B, and tangeretin administration in DSS treated murine UC. Surprisingly, some of them significantly increased overall richness and diversity while also regulating the composition of gut microbiota in a manner similar to the control group.

SCFAs, such as acetate, butyrate and propionate, are important energy sources and metabolites of intestinal microorganisms and colon epithelia cells, which exert remarkable action in preserving intestine homeostasis and strengthening gut barrier function (Feng et al., 2018; Venegas et al., 2019). Consistent with former researches, this research indicated that enteric SCFAs were reduced after DSS exposure, and baicalin and tangeretin administration could reverse these alterations through elevating the acetate, propionate, valeric and butyrate contents. Moreover, baicalin enriched certain specific bacteria populations that could motivate the generation of SCFAs, such as *Butyricimonas* spp., and *Lactobacillus* spp., manifesting that their protective action against DSS-evoked UC was mechanistically connected with elevating SCFAs generation mediated by their remodeling roles on the intestinal flora.

## Conclusion

**FIGURE 3:**
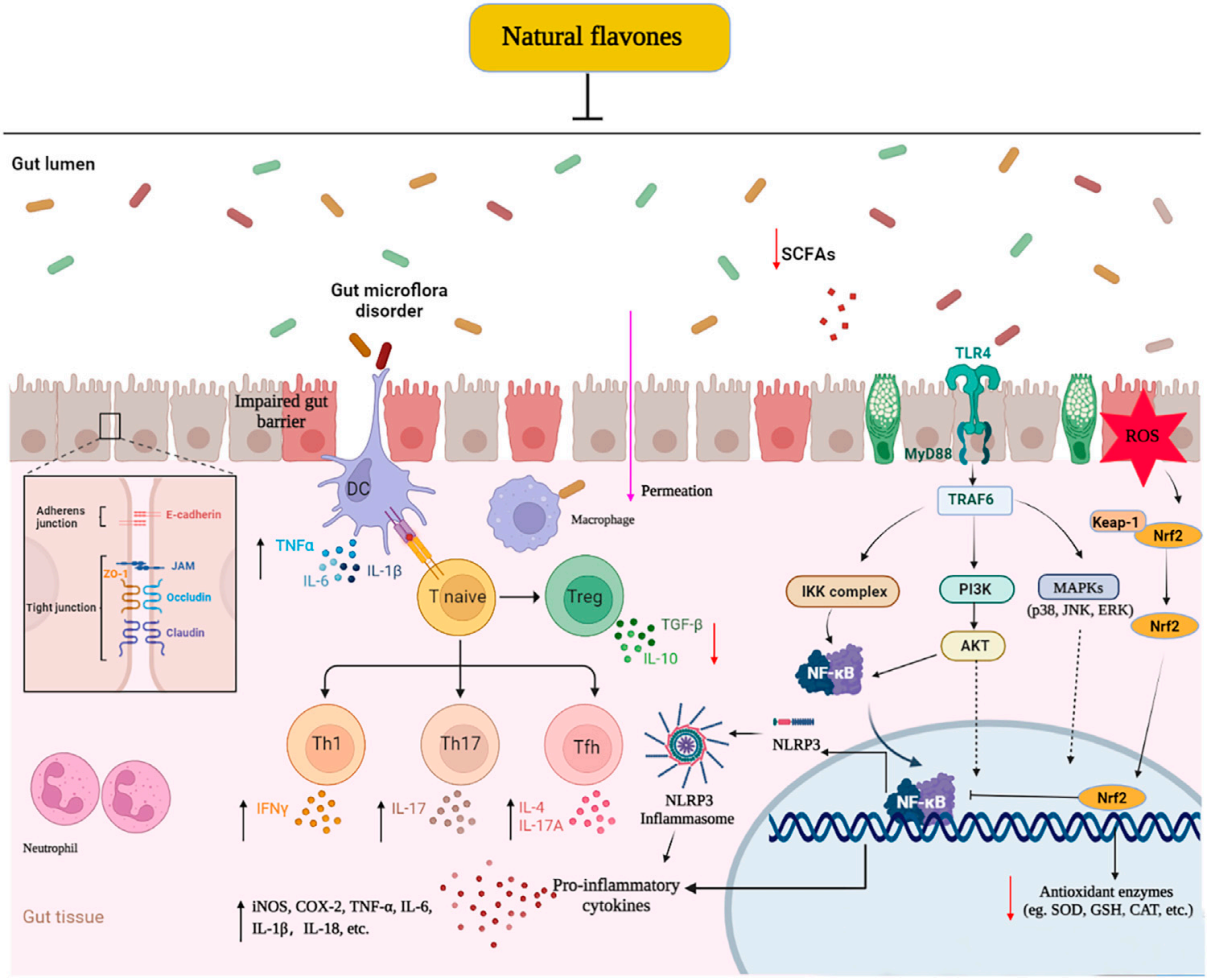
Molecular mechanisms of naturally derived flavones in the remedy of ulcerative colitis. ↓: Decrease, ↑: Increase: Inhibit.

This paper first generalizes the effective protection and potential mechanisms of natural flavones against UC *in vivo* and *in vitro* models. Their therapeutic roles manifested as the alleviation of clinical symptoms, relief of colonic mucosal injury, and inhibition of inflammatory responses. The potential mechanism of these effects mainly involves the blockage of various signalling pathways, including TLR4/MyD88/NF-κB, MAPKs, PI3K/AKT, and NLRP3 inflammasome, which regulate the intestinal microflora and Treg/Th17 balance, decreasing inflammatory responses and oxidative stress, and increasing TJ expression (Figure 3). The present review highlights the effects of flavones derived from natural sources in the treatment of UC by promoting gut homeostasis, improving intestinal barrier function, reducing oxidative stress, and regulating the immuno-inflammatory response. These findings revealed that these naturally derived flavones are effective and prospective drug candidates for UC and other inflammatory diseases. Whereas, the latent mechanism or toxicology of most natural flavones remains in-depth research, and their actual effect on colitis sufferers has not been verified in clinical experiments. Thus, more efforts should be focused to illuminating the molecular mechanisms and long-range effect and security in clinically relevant experiments to accelerate the perspective utilization of these flavones as colitis remedies in the near future.

## Figures and Tables

**Table:** 

**AA**	acetic acid
**Akt**	protein kinase B
**AMPK**	AMP-activated protein kinase
**Bcl-2**	B-cell lymphoma 2
**BMDMs**	bone marrow-derived macrophages
**CAT**	catalase
**Cl**	cleaved
**COX-2**	cyclooxygenase 2
**DAI**	disease activity index
**DCs**	dendritic cells
**DSS**	dextran sodium sulfate
**ERK**	extracellular signal-regulated protein kinase
**EZH2**	enhancer of zeste homolog 2
**Foxp3**	forkhead box P3
**GSH**	glutathione
**GSK-3β**	glycogen synthase kinase-3β
**GST**	glutathione S-transferase
**HMGB1**	high mobility group box protein 1
**HO-1**	heme oxygenase-1
**ICAM-1**	intercellular adhesion molecule-1
**IκBα**	inhibitor kappa B alpha
**IL**	interleukin
**iNOS**	inducible nitric oxide synthase
**IRAK1**	interleukin 1 receptor associated kinase 1
**LPS**	lipopolysaccharide
**MAPK**	Mitogen activated protein kinases
**MCP-1**	monocyte chemotactic protein-1
**MD-2**	myeloid diferentiation protein-2
**MDR1**	multidrug resistance 1
**MIP**	macrophage inflammatory protein
**MLCK**	myosin light chain kinase
**MMP**	matrix metalloproteinases
**MPO**	myeloperoxidase
**NF-κB**	nuclear transcription factor κB
**NLRP3**	NOD-like receptor 3
**NO**	nitric oxide
**NOD2**	nucleotidebinding oligomerization domain 2
**NOX4**	NADPH oxidases 4
**NQO-1**	NADP(H):quinone oxidoreductase 1
**Nrf2**	nuclear factor-erythroid 2-related factor 2
**PGE2**	prostaglandin E2
**PPAR-γ**	peroxisome proliferator-activated receptor γ
**p-STAT3**	phosphorylated signal transducer and activator of transcription 3
**PTEN**	phosphatase and tensin homolog
**PXR**	pregnane x receptor
**ROS**	reactive oxygen species
**RORγt**	tretinoic-acid-receptor-related orphan nuclear receptor gamma t
**SCFAs**	short-chain fatty acids
**SHP-1**	SH2-containing protein tyrosine phosphatase-1
**SOD**	superoxide dismutase
**SphK1**	sphingosine kinase 1
**S1PR1**	sphingosine 1-phosphate receptor 1
**TAK1**	transforming growth factor beta activated kinase 1
**TBARS**	thiobarbituric acid reactive substances
**TEER**	transepithelial electrical resistance
**TGF**	transforming growth factor
**TJ**	tight junction
**TJP1**	tight junction protein 1
**TLR4**	toll-like receptor 4
**TNBS**	24,6-trinitrobenzene sulfonic acid
**TNF**	tumor necrosis factor
**Treg**	regulatory T cell
**TXNIP**	thioredoxin-interacting protein
**UC**	ulcerative colitis
**ULK1**	UNC-51 like autophagy activating kinase 1
**JNK**	c-Jun N-terminal kinases
